# The Evolution of Medicine and Surgery for the past Thirty-five Years*Annual oration delivered at the Commencement of the Medical Department, University of Texas, June 1, 1910.

**Published:** 1910-07

**Authors:** A. W. Fly

**Affiliations:** Galveston, Texas, Member of Board of Regents, University of Texas


### TEXAS MEDICAL JOURNAL.

Established July, 1885

F. E. DANIEL, M. D., - - - - - - Editor, Publisher and Proprietor

Published Monthly.—Subscription $1.00 a Year.

Vol. XXVI AUSTIN, JULY, 1910. No. 1

The publisher is not responsible for the views of the contributors.

### Original Articles.

For Texas Medical Journal.

### The Evolution of Medicine and Surgery for the Past Thirty-five Years.*

A. W. FLY, M. D., GALVESTON, TEXAS, MEMBER OF BOARD OF REGENTS, UNIVERSITY OF TEXAS.

Thirty-five years ago I stood where these young graduates stand tonight, full of hope, vim, youth and ambition. In taking a restrospective glance at that time, it seems like an imperfect realization of a dim and distant dream. Had I the profound knowledge of a Gladstone, the sentimentality of a Cicero, the oratorical ability of a Demosthenes and the superb diction of a Prentiss, I could not adequately express to you the seriousness of this occasion.

In casting for a theme appropriate for this entertainment, I have concluded to invite your attention to the evolution of medicine and surgery for the past thirty-five years.

Throughout history the man of action comes first—and the fighting male, with his fists, his club, his battle-ax and his sword. After him, the poet, telling of his deeds. Then, after a long interval, the philosopher and the man of science. Reason and science do not belong to the days of legend. The history of medicine does not depart from the history of the people. The brilliant genius is the best example of his time.

*Annual oration delivered at the Commencement of the Medical Department, University of Texas, June 1, 1910.

We must expect to find the medical history of one era quite distinct from that of another. At first, the practice, remote and inadequate, of the Elizabethan England was brought here; then, with the succeeding century and the upgrowing generations of American-born men, came a wider knowledge, better general education and the first feeble attempt to combine wisdom and teaching of the old world with the changed experience of the new.

Today progress is the watchword of every true man of medicine. The open years of the twentieth century mark the highest degree of progress in the history of medicine. Congratulations are due the medical profession in that the science of medicine has kept pace with the other sciences, and today more than ever before the world realizes the fact that the practice of medicine is a science.

The work of recent years in surgery has been both voluminous and brilliant. The dawning of the new era of antisepsis and aspesis has been followed by startling achievements. The danger of sepsis and its correlating conditions, which before this time rose as a black nightmare before the vision of ambition, had limited surgery to a comparatively narrow part of the great science of healing. Before the immortal discoveries of the great Sir Joseph Lister, however, in one moment the forbidding walls were laid low. Fields hitherto looked upon as impossible now became the scene of busy activity in surgery. Pathological conditions which had previously baffled the wisest counsel and most ingenious devising of medicine now invited the efforts of the new surgery. Enthusiasm naturally ran wild, and with neither chart nor guide to control, experimental surgery began its new career. Before each aspiring surgeon lay open fields of unexplored wealth, with visions of fortune and eternal fame awaiting his command. Each man a pioneer, and none a guide, was truly an enticing opportunity. The work of this time was naturally, therefore, fraught with discovery more than at any time which had gone before, which perhaps gave it rank in history as the golden age of surgery. Many of its achievements have brought inestimable good to the human race and paved the way for surgery to its recognition as one of the grandest and most noble of all sciences and art.

In the remarkable advances of the last thirty years the thorax —that is, the firm walled cavity in which is inclosed the heart and lungs—has usually been considered beyond the realm of surgery. This impression is now being rapidly dispelled. Wounds of the heart, for instance, once thought to be inevitably fatal, are now within the proper domain of surgery. During recent years sutures have been inserted in the wounds of the heart in many cases. About one-half of the patients are still alive, with the repaired hearts in excellent condition. Even the lungs are no longer so puzzling to the profession. Operations on them are not only undertaken, but successfully carried out, and after-results in most cases very gratifying. Is it not encouraging to see with how much confidence medical men look upon tuberculosis, compared to a few years ago?

In abdominal surgery, perhaps, the most brilliant advances in operative work have been recorded. The surgery of this cavity has been simply marvelous, when we consider the short time in which it has all been accomplished. The invasion of the abdomen prior to the enunciation of the principle of antisepsis was a venture hazarded only under conditions of the most exacting necessity, and then with fear and trembling by the boldest of surgeons. How marvelous the change when now the operation is almost of daily occurrence in the practice of surgeons of eminence, and by many declared a safe and wise procedure even for exploration and diagnosis. Perhaps our enthusiasm has carried us beyond the limits of scientific rationalism and made surgery in some instances more a matter of mechanical dexterity than of mature judgment and wisdom. Yet the conscientious surgeon should not hesitate to enter the abdomen under any circumstances of accurate indication. Still he must, if he is true to the teachings of experience and honest in his relations to his reliant patient, remember that the opening of the abdominal cavity is not absolutely free from much danger, even in simple cases in the hands of trained operators, and under the strictest precautions. The old axiom, "Be sure you are right, then go ahead," is worthy of the scientific surgeon even today. Let us be thankful that the brain is recognized in surgery, and not the hand alone.

In looking backward, over the past thirty-five years, I find it replete with achievement—development and progress. An enumeration of the discoveries materially contributing to the science of medicine would fill many large volumes; but if it were required to indicate among all of these that one which, in the opinion of scientific men generally, has excited the most profound amazement and attracted the most widespread attention, we would, without a moment's hesitation, point to the discovery of Professor Roentgen of Wurzburg, Germany—the X-ray. It is singularly fitting that the achievements of the electrical world should thus have been crowned by the wonderful phenomena of the X-ray, just as the hands of the dial of time were about to point to the close of the century. To dwell upon its usefulness to the profession would be unnecessary.

Again, the educational acquirements of men in the medical profession, as well as in the other various businesses of life, have materially improved in the last thirty-five years. There was a time when the great majority of young men who received degrees at colleges and universities selected for their life work one of the learned professions. Such is not now the case. We find that since education is free the college-bred man is found in the counting rooms, banks, business houses, corporations, and, in fact, almost all vocations. Such men no longer look upon the doctor as omnipotent, but simply as a thinking, reasoning human being, whose profession is his business. And from my associations with investigators and scientific physicians, it is my observation that the most useful, and those who give us the most practical results, are men who investigate such difficult problems with the same care as other men consider difficult literary or business problems.

I have been in the practice of medicine for the past thirty-five years. I think I know what medicine was, in the past; I hope I know what medicine is, at the present; and if we are to believe what we read and what the world says we would suppose that medicine was newly made. The younger members of the profession, of course, are absorbed in the present—they know but little about the past, and it is very easy for us to persuade ourselves that there has been a sort of revolution in medicine; but if you stop to ask what this assumption is, you will find there is no revolution, there has simply been a change in the direction of thought—simply a change in the direction of the concerns of the medical mind for the well-being of those who look to medical men. The physician of the past was concerned in the disease, and not much concerned about the cause of the disease. The physician of the present is most concerned about the cause of the disease. The physician of the past struggled with disease—struggled to cure disease, without concerning much about the cause of the disease. Hence the invention of a term we are all familiar with: "Idiopathy." The strength of the physician of the past was idiopathy; the strength of the physician of the present is pathology. The physician of the past studied the nature of the disease; the physician of the present accepts the disease as a condition.

In the past there was just as much labor expended and just as much interest felt in medicine as at present, but, unfortunately, our energies were misdirected. Now all energy is utilized because we have abandoned certain chimerical notions, regarding vitality, regarding life, and we recognize them as a condition. And while the public would be ready, I suppose, if the present doctor were to tell them that he has learned that he can not cure a disease, to say: "Give us back the old doctor, who assumed that responsibility," and if the common mind were to know that all the expansiveness of surgery rests upon the fact that we have learned but to prevent disease, and know nothing more about the cure of them than those who preceded us they might be a little disappointed. But such is not the case. The modern doctor has found that the essential part of all disease is the cause; he has learned the habits of that cause; he has acquired knowledge that enables him to exclude that cause. So that what seems to be a revolution in medical thought is based upon facts that, if known by the general public, would modify very materially the estimate of the physician. And yet, it must be recognized as a great advancement. We must recognize this discovery of causes as the true goal of medical science, and I feel sure that as we progress we will reach a point where, realizing as distinctly as the physician of the past the incurability of disease, our hope will rest entirely upon the prevention of it.

Preventive medicine is to be the paramount issue, and the war cry of future medical thought. Who is to do this great work in the battle for humanity? The bacteriologist, the pathologist and the man of research go hand in hand. The psychologist must not be disregarded in this great work. He is now, and in the future is, to be reckoned with in the prevention of disease, and in the restoration from abnormal to normal conditions, both in mind and in body.

The specialist has come to stay. In the language of a great divine, the specialist is a man of high forehead, a French beard and a cold eye; the promoter of an impossibility; a leader of a forlorn hope. Then who is to assume this great responsibility?

The family physician, of course! The family physician, since the beginning of civilization, is so important, such an integral part of society, that, like Voltaire's god, if he did not exist, he would have to be created.

Who have been some of the leading lights, representing the family physician, in the past? One hundred and one years ago this month Ephraim McDowell, of Danville, Ky., an obscure physician, did, by his fertile brain, conceive the idea, and his bold hand execute an operation that has become a recognized surgical procedure throughout the civilized world—is now of daily occurrence and has saved hundreds of thousands of human lives.

Crawford Long, of Georgia, in 1842, administered ether for the purpose of doing a surgical operation—two years before Wells, the dentist of Boston (who has received the honors, unjustly bestowed, for this invention).

J. Marion Sims, another obscure physician, devised an operation and made successful, by its performance, the possibility of relieving woman of the most loathsome disease known to her sex.

But to return home: It was Greenville Dowell, of Galveston, who first suggested the mosquito theory of yellow fever.

W. D. Kelley, of Galveston, was the first to claim that tuberculosis was both infectious and contagious, long before Pasteur and Koch were dreamed of as intellectual possibilities.

Last, but by no means least, is our old friend, J. F. Y. Paine, who has continuously taught obstetrics and gynecology for thirty years, nineteen of which have been spent with the Medical Department of the University of Texas. He retires of his own volition at three score years and ten, full of honors and with the united love of his friends and colleagues, a man whose record is as bright as the driven snow, whose ideals are as lofty as the mountain height, a man who has been faithful to every obligation, true to every trust and loyal to every condition in life.

The family physician is not a man to be disregarded in any relation in life. The man who stands on the pilot bridge, when the great ship of nature is seeking a haven of maternal rest, and guides her between the shoals; when motherhood is struggling with the cruel waves of fate, and makes a safe landing, is not a fit subject for oblivion; the man who cares for you in infancy, looks after you in childhood and tenders you the proper advice in young manhood or young womanhood, can not be wholly ignored as beyond the pale of faithful consideration.

As time goes on and as civilization advances, the work of the family physician will be better known, and more appreciated; in fact, when he shall have ceased to be an earthly factor his memory will stand out like a great oak, in the forest of life.

Death's cold, white hand is like the snow laid softly on the furrowed hill;
It hides the broken seams below, and leaves the summit brighter still.

